# CoryneRegNet 4.0 – A reference database for corynebacterial gene regulatory networks

**DOI:** 10.1186/1471-2105-8-429

**Published:** 2007-11-06

**Authors:** Jan Baumbach

**Affiliations:** 1 Computational Methods for Emerging Technologies, Bielefeld University, Bielefeld, Germany; 2 International Graduate School in Bioinformatics and Genome Research, Center for Biotechnology, Bielefeld, Germany

## Abstract

**Background:**

Detailed information on DNA-binding transcription factors (the key players in the regulation of gene expression) and on transcriptional regulatory interactions of microorganisms deduced from literature-derived knowledge, computer predictions and global DNA microarray hybridization experiments, has opened the way for the genome-wide analysis of transcriptional regulatory networks. The large-scale reconstruction of these networks allows the in silico analysis of cell behavior in response to changing environmental conditions. We previously published CoryneRegNet, an ontology-based data warehouse of corynebacterial transcription factors and regulatory networks. Initially, it was designed to provide methods for the analysis and visualization of the gene regulatory network of *Corynebacterium glutamicum*.

**Results:**

Now we introduce CoryneRegNet release 4.0, which integrates data on the gene regulatory networks of 4 corynebacteria, 2 mycobacteria and the model organism *Escherichia coli K12*. As the previous versions, CoryneRegNet provides a web-based user interface to access the database content, to allow various queries, and to support the reconstruction, analysis and visualization of regulatory networks at different hierarchical levels. In this article, we present the further improved database content of CoryneRegNet along with novel analysis features. The network visualization feature GraphVis now allows the inter-species comparisons of reconstructed gene regulatory networks and the projection of gene expression levels onto that networks. Therefore, we added stimulon data directly into the database, but also provide Web Service access to the DNA microarray analysis platform EMMA. Additionally, CoryneRegNet now provides a SOAP based Web Service server, which can easily be consumed by other bioinformatics software systems. Stimulons (imported from the database, or uploaded by the user) can be analyzed in the context of known transcriptional regulatory networks to predict putative contradictions or further gene regulatory interactions. Furthermore, it integrates protein clusters by means of heuristically solving the weighted graph cluster editing problem. In addition, it provides Web Service based access to up to date gene annotation data from GenDB.

**Conclusion:**

The release 4.0 of CoryneRegNet is a comprehensive system for the integrated analysis of procaryotic gene regulatory networks. It is a versatile systems biology platform to support the efficient and large-scale analysis of transcriptional regulation of gene expression in microorganisms. It is publicly available at .

## Background

Recently several whole-genome sequencing projects have generated huge amounts of data related to various microorganisms including gene and protein sequences and their functional annotations. These annotations can be performed, stored and analyzed with tools like e.g. GenDB [1].

In order to handle changing environmental circumstances and to maintain growth and survival, the genes activity varies under different conditions. One major goal in systems biology is to understand the process of their transcriptional regulation. The application of post-genomic analysis techniques to bacterial genome sequences provides knowledge to encoded proteins involved in the gene regulation. Microarray experiments can be used to study the expression of genes and the results can be stored and analyzed using tools like EMMA [2]. This data along with literature-derived knowledge on the regulation of gene expression has opened the way for genome-wide reconstruction of transcriptional regulatory networks. These large-scale reconstructions can be converted into in silico models of corynebacterial cells that allow systematic analysis of network behavior in response to changing environmental conditions. [3-7]

Besides pathogenic corynebacterial species of medical importance, like *C. diphtheriae *and *C. jeikeium*, other corynebacteria like *C. glutamicum *and *C. efficiens *are traditionally used in biotechnological production processes. We previously designed CoryneRegNet, which is an ontology-based data warehouse implemented to facilitate the genome-wide reconstruction of transcriptional regulatory networks of corynebacteria and *Escherichia coli*. [8,9] It is based on a multi-layered, hierarchical and modular concept of transcriptional regulation and was implemented by using an ontology-based data structure. We integrated a fast and statistically sound method (PoSSuMsearch [10]) to predict transcription factor binding site motifs within and across species. It is the only available software package that is fast enough to provide interactive response times for large-scale PSSM searches and at the same time integrates exact statistics for p-value computations. Reconstructed regulatory networks can be visualized on a web interface and as graphs. Special graph layout algorithms have been developed and implemented to facilitate the comparison of gene regulatory networks across species. This is can be very benefitial for studying gene regulatory networks, as shown e.g. in [11-13].

A related system is RegulonDB [14]. It focuses on *E. coli*, and so far lacks tools essential for regulatory network reconstruction and analysis such as binding site motif matching, protein cluster calculation, and homology detection. It furthermore just provides very simple network visualization and no network comparison and analysis features. Another existing system is PRODORIC [15], which has a comprehensive goal, but for most procaryotes only contains the available NCBI genome annotation and no or just little gene regulatory data. Further information on gene regulations is available about *E. coli *(as in RegulonDB), *B. subtilis*, and *P. aeruginosa*. It does not provide the network visualization and comparison capabilities of CoryneRegNet, and its motif matching tools are based on a similarity score, not statistical considerations. Both, PRODORIC and CoryneregNet offer genome browsers. But PRODORIC also provides gene ontology (GO) links, and some gene expression profiles (stimulons), all of which are yet to be integrated into CoryneRegNet.

A prerequisite to systems biology is the integration of heterogeneous experimental and annotation data, which are stored in numerous life-science databases. Efficient data handling and integration is prevented by a wide range of problems. The major problem, however, is the querying procedure, since it requires detailed semantic knowledge about the content of specific database tables. [16] In the case of the above mentioned systems GenDB and EMMA, a special interface called BRIDGE [17,18] has been developed, to overcome these data exchange problems. A more general and more widely used and accepted technique is the application of SOAP based Web Services. These have recently been implemented e.g. by the European Bioinformatics Institute [19], or BRENDA [20].

In this article, we describe, how CoryneRegNet has been improved to provide the following updated database content and analysis features:

1. Integrated Web Service clients for GenDB and EMMA provide further gene annotation data and stimulon (microarray) data respectively.

2. A publicly available Web Service server offers methods to other bioinformatics applications to query data from CoryneRegNet.

3. Protein clusters are integrated by using the heuristic cluster editing software FORCE.

4. Integration of corynebacterial stimulon data.

5. Detection of putative contradictions in microarrays based on known gene regulatory networks and predicted operons (COMA feature).

First, the updated system architecture is briefly described followed by the integration of the above listed features. Subsequently, the improvements of CoryneRegNet are discussed. We exemplarily show the integrated usage of the EMMA Web Service and summarize the updated database content. The COMA feature is illustrated by means of an artificial example. In the last section, we sum up the article and conclude with how the community can profit from CoryneRegNet 4.0

## Construction and content

### System architecture

**Figure 1:**
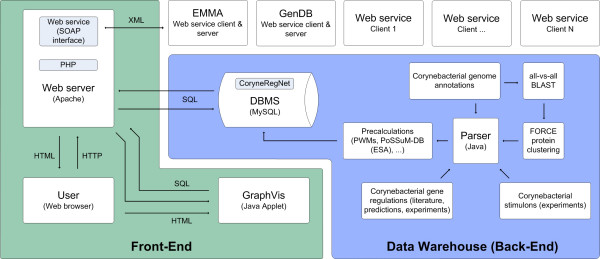
**System architecture of CoryneRegNet**. This figure illustrates the system architecture of CoryneRegNet 4.0. It is a data warehouse: All time-consuming calculations are performed at data warehousing. The results are then transformed into an ontology-based data structure and imported to the MySQL database server (Back-End). An Apache web server processes the user requests, queries the database and constructs the corresponding web pages. It further provides the SOAP based Web Service servers for GenDB, EMMA, and other applications and also queries them as a client. A Java Applet is used for network visualization and analysis (Front-End).

In order to integrate Web Services and protein clusters, the architecture of CoryneRegNet has been changed. Figure 1 illustrates the system architecture of the CoryneRegNet release 4. Since it is a data warehouse, all time-consuming calculations (all-vs-all BLAST, protein cluster computations, construction of the PoSSuMsearch enhanced suffix array) are regularly performed at import process. The results are TAB-delimited flat files, which subsequently are transformed into an ontology-based data structure, which mainly consists of typed concepts and relations. This has been described previously in [8,21,22]. The transformed data is then imported into a MySQL 4.1.9 database server [23]. An Apache HTTP server 2.0.49 [24] processes the user requests, queries the database and constructs the corresponding web pages, by using PHP 5.2.1 [25]. It further provides the SOAP based Web Service servers for GenDB, EMMA, and other applications and also queries them as a client. The Java Applet GraphVis is used for network visualization and analysis (at least Java version 1.4.2 is necessary, [26]).

### Web service integration

#### Clients for GenDB and EMMA

Since both, GenDB and EMMA are implemented in Perl, they provide servers utilizing the SOAP::Lite library [27]. In CoryneRegNet, the clients as well as the server are implemented by using NuSOAP 0.7.2 for PHP [28].

CoryneRegNet profits on several aspects from the direct connection to GenDB and EMMA:

• To a gene of interest, more accurate and up to date annotation data from GenDB is displayed in the detailed view of a gene (EC numbers for enzymes, Gene Ontology numbers, etc.).

• Using the GenDB Web Service, all target genes of a transcription factor are linked to KEGG pathways [29] and a list of regulated pathways is presented. This allows insights into the general nature of a transcription factor.

• The build-in network visualization Applet GraphVis now features the projection of stimulon data (gene expression levels) extracted from EMMA to the size of the concerned nodes, which represent the genes.

Normally, this kind of interconnections are realized by HTML-links to other web pages or by regular, manual downloads and a subsequent integration of the corresponding data. Using SOAP based Web Services, the user even does not recognize, that the data is downloaded from another service. Furthermore, the data can be post-processed easily and hence presented in a different way, and, most important, it is always up to date.

### Server

The publicly available Web Service server offers several methods to query data from CoryneRegNet. Neither direct access to the MySQL server is necessary, nor any knowledge about the data structure in the back-end. All API information is provided as WSDL file. The WSDL file for the interface of CoryneRegNet is automatically generated on demand by that PHP/SOAP script, which also implements the Web Service functions. After a requirements analysis with biologists, who use CoryneRegNet, we decided to provide the following methods:

• getOrganisms: Compares a given string to all organism names in the database and returns for all matches the unique organism identifier.

• getTfGeneIDs: Returns all identifiers of genes that code for transcription factors, for a given organism ID.

• getGeneID: Genes can have ambiguous IDs in different databases and most of them are additionally stored in CoryneRegNet. This methods returns unique internal gene IDs, given an ambiguous one.

• regulates: For a given gene X, all genes that are regulated by X are returned, including additional information (evidence, regulation type, PubMedID, etc.).

• isRegulatedBy: For a given gene X, all genes that regulate X are returned, including additional information.

• getOperonByGeneID: Returns operon information for a given gene ID.

Now, it is possible to retrieve the most important data from CoryneRegNet directly from any software that is written in a programming language, which offers a SOAP interface. Such a program can internally handle all queried data as if the data would be stored in local data structures and memory.

A detailed documentation (with examples) on how to implement a CoryneRegNet Web Service client is available at the CoryneRegNet 4.0 web site. More general information on Web Services and SOAP can be found in [30,31]

### Protein cluster integration with FORCE

We recently developed FORCE, an algorithm that heuristically solves the weighted graph cluster editing problem (WGCEP). Given a symmetric, pairwise similarity function *s*(*x*, *y*), that assigns a similarity score to every pair of objects *x, y *of a set, one can imagine a graph. The objects are represented as nodes. Every node *x *has an undirected edge to every other node *y *in the graph with edge weight *s*(*x*, *y*). Given a threshold *t*, an edge is present, if *s*(*x*, *y*) > *t*, and it is not present, if *s*(*x*, *y*) ≤ *t*. We now can define costs *c*(*x*, *y*) for modifying edges, as *c*(*x*, *y*) = |*t *- *s*(*x*, *y*)|. We further need the following definition:

**Definition 1. **An undirected simple graph *G *= (*V*, *E*) is called *transitive *if

For all triples uvw∈(V3),uv∈E and vw∈E implies uw∈E.
 MathType@MTEF@5@5@+=feaafiart1ev1aaatCvAUfKttLearuWrP9MDH5MBPbIqV92AaeXatLxBI9gBaebbnrfifHhDYfgasaacPC6xNi=xI8qiVKYPFjYdHaVhbbf9v8qqaqFr0xc9vqFj0dXdbba91qpepeI8k8fiI+fsY=rqGqVepae9pg0db9vqaiVgFr0xfr=xfr=xc9adbaqaaeGacaGaaiaabeqaaeqabiWaaaGcbaqbaeqabeGaaaqaaiabbAeagjabb+gaVjabbkhaYjabbccaGiabbggaHjabbYgaSjabbYgaSjabbccaGiabbsha0jabbkhaYjabbMgaPjabbchaWjabbYgaSjabbwgaLjabbohaZjabbccaGiabdwha1jabdAha2jabdEha3jabgIGiopaabmaabaqbaeqabiqaaaqaaiabdAfawbqaaiabiodaZaaaaiaawIcacaGLPaaacqGGSaalaeaacqWG1bqDcqWG2bGDcqGHiiIZcqWGfbqrcqqGGaaicqqGHbqycqqGUbGBcqqGKbazcqqGGaaicqWG2bGDcqWG3bWDcqGHiiIZcqWGfbqrcqqGGaaicqqGPbqAcqqGTbqBcqqGWbaCcqqGSbaBcqqGPbqAcqqGLbqzcqqGZbWCcqqGGaaicqWG1bqDcqWG3bWDcqGHiiIZcqWGfbqrcqGGUaGlaaaaaa@6CBB@

A transitive graph is a union of disjoint cliques, i.e., of complete subgraphs. Each clique represents, in our case, a protein cluster. Since the initial graph, derived from protein similarity values, may not be transitive, we need to modify it. The goal is to modify a given intransitive graph by adding/removing edges in such a way, that the graph is transitive with minimal costs for modifications. This strategy can be used to cluster elements together. Unfortunately, the WGCEP is NP-hard.

In [32], we presented a Fixed Parameter algorithm, that solves the WGCEP for connected components with up to approx. 60 nodes. Since, in the case of CoryneRegNet, one has to cluster more then 20,000 protein sequences and connected components with more then 500 nodes, we also developed a heuristic, that provides very close to optimal solutions in polynomial time: FORCE. The main idea is based on a force-based graph layouting algorithm, initially introduced by Fruchterman and Reingold [33]. The algorithm has been modified with respect to the edge weights to group highly-interconnected subgraphs closer together than others. Afterwards, FORCE uses Single Linkage Clustering to retrieve a clustering and Restricted Neighborhood Search Clustering [34] to reduce the number of singletons and further improve the result. The software and with the clustering results are available at [35].

For the clustering of protein sequences, FORCE first uses all-vs-all BLAST results of all protein sequences of all organisms in CoryneRegNet as input. In the next step, the heuristic is applied and the clustering results are written into a TAB-delimited flat file and subsequently imported into the database back-end (also refer Figure 1, right).

### Stimulon integration

If a microarray experiment has been performed in wet lab, EMMA can be used for storing and analyzing the results. The Web Service client of CoryneRegNet can be used for the projection of the gene expression levels to a visualized gene regulatory network to check for consistency with known regulatory pathways and to gain new insights. Beside the possibility to use unpublished, short-dated and often transient expression data from EMMA, we additionally imported verified and published corynebacterial stimulon data directly into the CoryneRegNet database. A stimulon is a set of genes and we integrated that genes where the M-value *|m| > *1. Table 1 summarizes the available experiments. Further microarray results can be included easily upon request.

**Table 1:** Stimulons directly integrated in CoryneRegNet 4.0

Organism	Short description	Nr. of genes	Publication
*C. glutamicum*	ΔDtxR (cg2103) vs. wildtype	255	[38]
*C. glutamicum*	ΔLtbR (cg1486) vs. wildtype	50	[39]
*C. glutamicum*	ΔMcbR (cg3253) vs. wildtype	134	[40]
*C. glutamicum*	ΔSigM (cg3420) vs. wildtype	37	[41]
*C. glutamicum*	ΔSsuR (cg0012) vs. wildtype	29	[42]
*C. glutamicum*	grown on acetate/propionate vs. acetate	160	[43]
*C. glutamicum*	res167 transition vs. res167 exponential	111	[44]
*C. jeikeium*	wildtype vs. wildtype + vanillylalcohol	93	[45]

This table briefly summarizes the stimulons that are integrated in CoryneRegNet.

### The COMA feature

The COMA feature is a novel option in the CoryneRegNet front-end to facilitate consistency checks in microarrays with known regulatory networks. The user has 3 possibilities to enter gene expression data:

• Copy+paste into a text field.

• Upload a TAB-delimited flat file.

• Usage of stimulon data from the CoryneRegNet database.

We denote an upstimulation with '+' and a downstimulation with '-' respectively. The same can be done to an activation or repression of a target gene by a regulator. Let *g *∈ {+, -} be the stimulation state of a gene *G*. Let *t *∈ {+, -} be the stimulation state of the transcription factor *T*, which regulates *G*. Let *r *∈ {+, -} be the type of the known regulation of *G *by *T*. Now consider the algebraic signs in the following equation: *t*·*g *= *r*. If the equation is incorrect (e.g. '+'·'-' = '+') we define this as an inconsistency. Following this, for every gene *G *of a given microarray experiment with expression state *g*, CoryneRegNet queries the database and retrieves all transcription factors *T*, which regulate *G*.

Subsequently, we check for all transcription factors the expression state *t *and the regulation relationship *r *and apply the above explained inconsistency test. For every inconsistent measurement, we also report, if other transcription factors regulate the gene *G *and hence possibly could explain the inconsistent expression level. Furthermore, we test, if all genes within all predicted operons in CoryneRegNet are regulated identical (all '+' or all '-') and report them otherwise.

**Figure 2:**
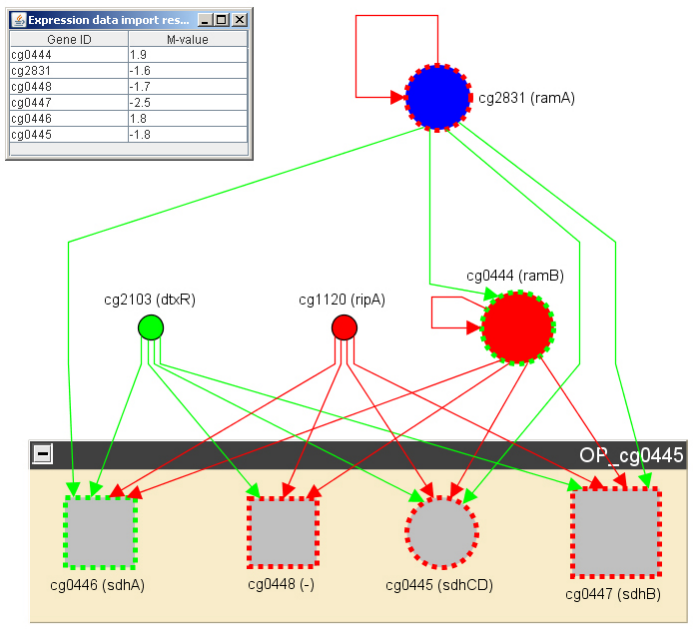
**An artificial stimulon**. This screenshot shows the improved network analysis and visualization feature GraphVis. Presented is the artificial stimulon of table 2 projected onto the underlying gene regulatory network. The nodes represent genes and the edges gene regulations. Red nodes are repressors, green nodes activators, and blue nodes dual regulators. Gray nodes are target genes. A red edge represents a repression, a green edge an activation, and a blue edge a sigma factor regulation. The nodes sizes are relative to the expression value (M-value): the bigger the node, the more the differential expression of the respective gene. Genes can be upstimulated (green dotted node border) or downstimulated (red dotted border). The big multi-node groups genes to an operon. The circular node inside the operon is that gene, which is preceded by a transcription factor binding site.

**Figure 3:**
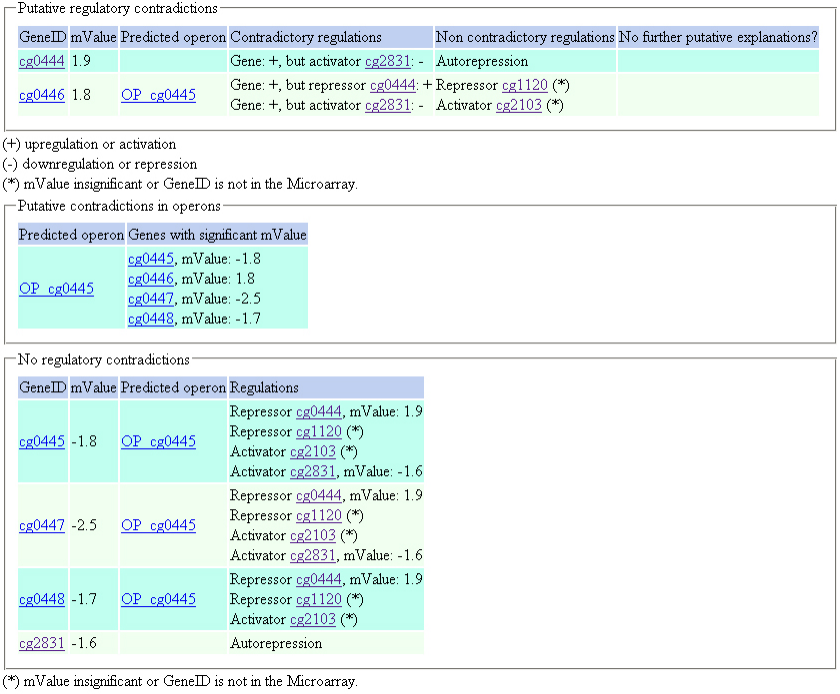
**Result of the COMA feature applied to an artificial stimulon**. This screenshot shows the result page of the COMA feature if applied to the artificial stimulon of table 2. There are 3 putative contradictions: 2 for *sdhA *(cg0446) and 1 for *ramB *(cg0444). For both genes, there are further transcriptional regulators listed that possibly could resolve the contradictions.

For simplification, we explain this by means of an artificial example. Consider the small stimulon experiment in Table 2 (and in Additional File 1) and its visualization in Figure 2. One can see 3 putative contradictions: (1) The gene *sdhA *is upregulated, while all the other genes in the same predicted operon are downregulated. (2) The gene *ramB *is upregulated, but the activator *ramA *is downregulated. (3) The gene *sdhA *is upregulated, while the activator *ramA *is downregulated and the repressor *ramB *is upregulated. If these experimental results are copy+pasted into the COMA feature input textfield, we retrieve the same results automatically (refer the screenshot shown in Figure 3). For such an expression profile, one would argue if something went wrong with the experiment, or if there are further yet unknown regulatory interactions. It is obvious, that the method also helps with the improvement of the predicted operons.

**Table 2:** Artificial corynebacterial stimulon

Gene	GeneID	Operon	M-value	Regulated by
*ramB*	cg0444	-	1.9	(R) *ramB*, (A) *ramA*
*sdhCD*	cg0445	*OP_cg*0445	-1.8	(R) *ramB*, (R) *ripA*, (A) *dtxR*, (A) *ramA*
*sdhA*	cg0446	*OP_cg*0445	1.8	(R) *ramB*, (R) *ripA*, (A) *dtxR*, (A) *ramA*
*sdhB*	cg0447	*OP_cg*0445	-2.5	(R) *ramB*, (R) *ripA*, (A) *dtxR*, (A) *ramA*
-	cg0448	*OP_cg*0445	-1.7	(R) *ramB*, (R) *ripA*, (A) *dtxR*, (A) *ramA*
*ramA*	cg2831	-	-1.6	(R) *ramA*

This table shows a small, artificial stimulon, which can be applied to the novel consistency check feature of CoryneRegNet 4.0. Expression values are given as M-values. In the last column, we list that transcription factors, which control the gene in the first column. We denote (R) as repression and (A) as activation respectively. One can see 3 putative contradictions: (1) The gene *sdhA *is upregulated, while all the other genes in the same predicted operon are downregulated. (2) The gene *ramB *is upregulated, but the activator *ramA *is downregulated. (3) The gene *sdhA *is upregulated, while the activator *ramA *is downregulated and the repressor *ramB *is upregulated. Refer figure 3 for a visualization.

## Utility and Discussion

### Database content development

Table 3 summarizes the development of the database content of CoryneRegNet from the first release 1.0 to the actual version 4.0. One can see, that beside novel visualization and analysis features, also the amount of available data increased continuously.

**Table 3:** Database content development of CoryneRegNet

Ver.	Org	Genes	TFs	Reg. genes	Regs	BM	PWM	Stim	Clust
1.0	1	3058	53	331	430	192	23	-	-
2.0	4	10432	64	499	607	274	29	-	-
3.0	5	14737	213	1632	2912	1522	130	-	-
4.0	7	22920	213	1632	2912	1522	130	8	4548

This table summarizes the development and growth of CoryneRegNet from the first release 1.0 to the actual version 4.0. Legend: Ver = CoryneRegNet version, Org = organisms, Genes = genes, TFs = regulators, Reg. genes = regulated genes, Regs = regulations, BM = binding motifs, PWM = position weight matrices, Stim = stimulons, Clust = protein clusters.

At the Center for Biotechnology at Bielefeld University, we still perform experiments with corynebacteria and now also with *Mycobacterium tuberculosis*. CoryneRegNet is used to predict gene regulatory networks for mycobacteria mainly based on the knowledge from *C. glutamicum*. Therefor, the genomes of *Mycobacterium tuberculosis CDC1551 *and *Mycobacterium tuberculosis H37Rv *have been included into CoryneRegNet 4.0. The database content will be updated as soon as novel and experimentally verified data is available. Since we already did this continuously in the past, the number of regulations, regulators, binding motifs, etc. for the releases 3.0 and 4.0 do not differ in the last two rows of Table 3.

### Novel data presentation, analysis, and visualization features

The Web Service client for GenDB now allows the presentation of more up to date gene annotation data for a displayed gene at the CoryneRegNet website. Listed are a description, comments, and an assigned function with evidence and confidence. Furthermore, an EC number for enzymes, COG numbers (with links to COG [36]), and GO numbers (with links to Gene Ontology [37]) are presented. All target genes of a transcription factor are additionally mapped to KEGG pathways (with links to KEGG). Hence, a list of regulated pathways can be presented.

**Figure 4:**
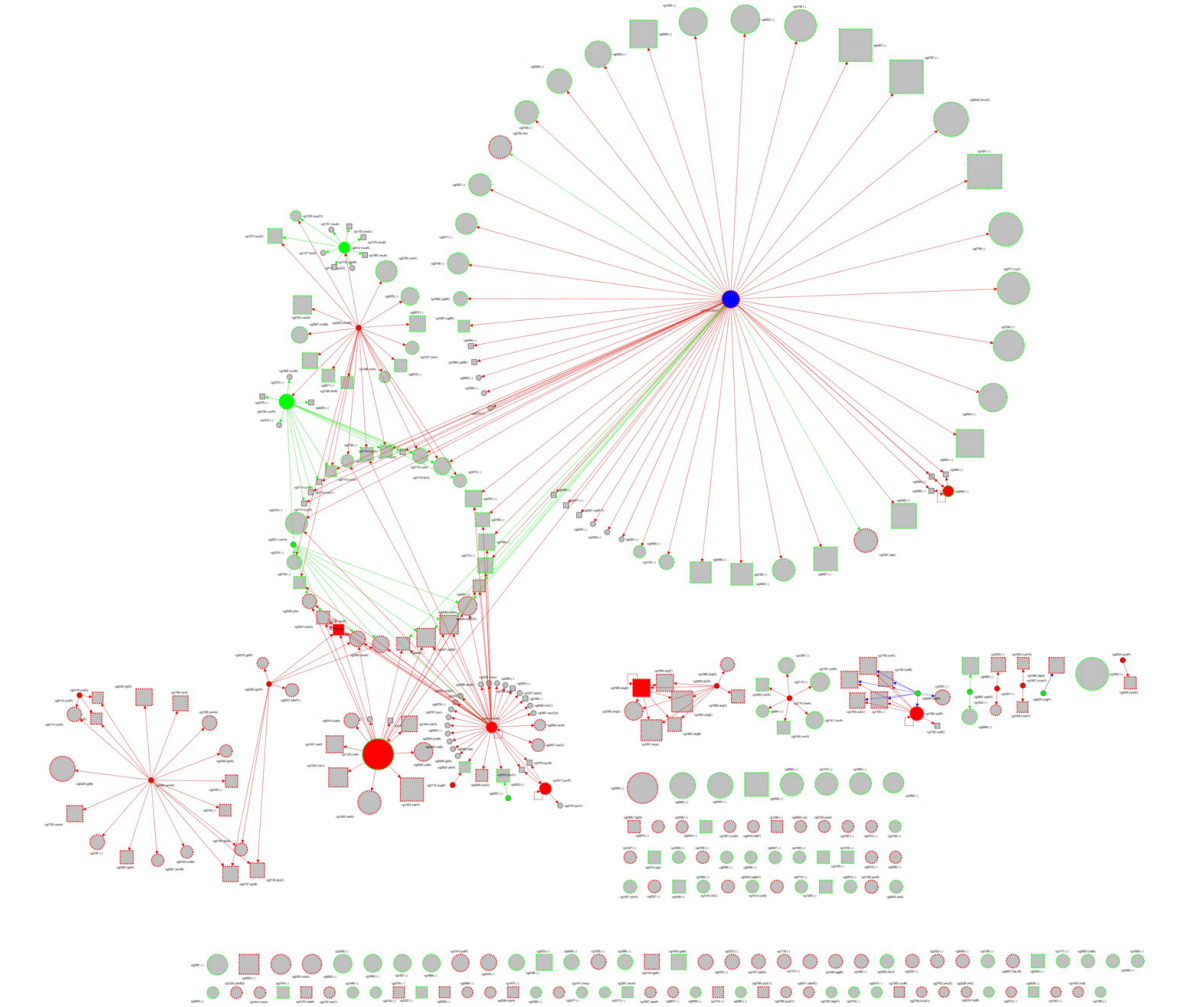
**The ΔDtxR stimulon**. This screenshot of the GraphVis feature of CoryneRegNet shows all the genes that are simulated by the ΔDtxR stimulon and all known corresponding transcriptional regulatory interactions. Also refer the legend of figure 3 for color codes.

The Web Service client for EMMA provides data on gene expression. Further stimulons have been directly integrated into the CoryneRegNet database back-end. Figure 2 and Figure 4 exemplarly show the improved network visualization toolkit GraphVis. Displayed are all the genes that are stimulated by the artificial stimulon presented in Table 2 (Figure 2) and the ΔDtxR stimulon (Figure 4). The nodes represent genes and the edges transcriptional regulatory interactions. The user can zoom into the graph, apply different graph layout styles, extend the graph dynamically with more genes/regulations from the database, and apply further gene expression data from EMMA, the stimulon repository of CoryneRegNet, or from own text- or MS-Excel files.

The Web Service server now allows other software developers to integrate CoryneRegNet data directly into their own software. Just a SOAP interface for the respective programming language and an internet connection are necessary. Common practice is to provide flat files for download (as e.g. in RegulonDB), which are updated in regular time intervals. Hence the data often is out of date and has to be transformed into a data structure compatible with the corresponding programming language. With SOAP-based Web Services, CoryneRegNet 4.0 overcomes these disadvantages.

The integrated COMA feature facilitates with consistency checks in microarray results. The method provides hints for incorrectly predicted operons, missing gene regulatory interactions and putative errors in the experimental results. Microarray results can be uploaded or copy+pasted easily and subsequently are checked for consistency with the known gene regulations of the integrated corynebacteria, mycobacteria, and *Escherichia coli*.

## Conclusion

Novel ultra-fast sequencing and large-scale postgenomic analysis techniques of complete genome sequences recently generated a vast amount of data that has to be analyzed. Comprehensive evaluation of that data asks for user-oriented software platforms supporting data management, visualization, integration into existing knowledge, generation of hypotheses, evaluation, and the possibility to share this post-processed data with others.

With release 4.0, CoryneRegNet now is a comprehensive system for the integrated analysis of procaryotic gene regulatory networks. The database contains information on DNA-binding transcription factors and on transcriptional regulatory interactions of corynebacteria, mycobacteria and *E. coli*. The results of global DNA microarray hybridization experiments have been integrated as stimulons into the CoryneRegNet data repository. A web-based user interface provides access to the database content, allows various queries and supports the reconstruction, visualization, validation and prediction of regulatory networks at different hierarchical levels. CoryneRegNet is moreover linked to several databases (EMMA, GenDB, COG, GO, NCBI, etc.). Although CoryneRegNet initially was developed as a data warehouse of transcriptional regulatory networks of *C. glutamicum*, its ontology-based design along with its programs and scripts has been designed for a general applicability to other species. Hence, it has been extended with genomic and transcriptional data on 6 more organisms, experimental results (stimulons) and computer predictions (protein clusters, PWM-based binding motif predictions, etc.). As it is state-of-the-art, CoryneRegNet is connected to other data sources using Web Services and provides an own Web Service for external consumers. Consequently, CoryneRegNet 4.0 is a versatile systems biology platform to support the efficient and large-scale analysis of transcriptional regulation of gene expression in microorganisms.

## Availability and Requirements

Project name: CoryneRegNet 4.0

Project home page: 

Operating system(s): Platform independent

Programming language: PHP, Java 6

License: Academic Free License (AFL)

Any restrictions to use by non-academics: No.

Comment: A documentation on how to develop a CoryneRegNet Web Service client is available at the web site. Instructions on how to adapt the data from other microorganisms for the integration and the regulatory analysis within CoryneRegNet is available upon request.

## Authors' contributions

JB designed and implemented CoryneRegNet 4.0.

## Supplementary Material

Additional file 1 Artificial stimulon. This file is a tab-delimited flat file that stores the artificial stimulon explained in table 2. The file can directly be submitted to the COMA feature of CoryneRegNet 4.0 or projected e.g. onto the *ramA*/*ramB *network if visualized in GraphVis. Click here for file
